# Can hair concentrations of artemether-lumefantrine be used as a tool to retrospectively determine drug exposure during malaria treatment?

**DOI:** 10.1016/j.nmni.2025.101617

**Published:** 2025-07-29

**Authors:** Jenny L. Schnyder, Marloes Vos-van der Meer, Reinier M. van Hest, Ron Mathot, Patricia Schlagenhauf, Hanna K. de Jong, Martin P. Grobusch

**Affiliations:** a Center for Tropical Medicine and Travel Medicine, Department of Infectious Diseases, Division of Internal Medicine, Amsterdam UMC, Location University of Amsterdam, Amsterdam, Netherlands; b Department of Hospital Pharmacy & Clinical Pharmacology, Amsterdam UMC, Location University of Amsterdam, Amsterdam, Netherlands; c University of Zurich Centre for Travel Medicine, WHO Collaborating Centre for Travelers' Health, Department of Public and Global Health, Military Medicine Biology Competence Centre, Institute for Epidemiology, Biostatistics and Prevention, Zurich, Switzerland; d Institute of Tropical Medicine, German Centre for Infection Research (DZIF), University of Tübingen, Tübingen, Germany; e Centre de Recherches Médicales en Lambaréné (CERMEL), Lambaréné, Gabon; f Instituto de Microbiologia, Faculdade de Medicina, Universidade de Lisboa, Lisbon, Portugal; g Masanga Medical Research Unit (MMRU), Masanga, Sierra Leone; h Institute of Infectious Diseases and Molecular Medicine (IDM), University of Cape Town, Cape Town, South Africa

**Keywords:** Malaria, Artemether-lumefantrine, Antimalarials, Recrudescence, Hair, Travelers

## Abstract

**Background:**

Artemether-lumefantrine (AL) is the first-line treatment for uncomplicated *Plasmodium falciparum* malaria, with cure rates exceeding 95 %. However, recrudescence occurs in 2–14 % of cases, often linked to inadequate lumefantrine exposure. Retrospective assessment of drug exposure in recrudescence cases is challenging, as lumefantrine levels are undetectable in blood after several weeks. Hair analysis may offer an alternative method to assess drug exposure over time. The objective of this proof-of-concept study was to assess whether artemether and lumefantrine, and their respective metabolites dihydroartemisinin and desbutyl-lumefantrine, could be detected and quantified in hair of falciparum malaria patients who completed an AL treatment course.

**Methods:**

Hair samples were collected from six patients with falciparum malaria at Amsterdam UMC, four weeks after treatment initiation. Samples were analysed using liquid chromatography-tandem mass spectrometry (LC-MS/MS).

**Results:**

Lumefantrine and its metabolite desbutyl-lumefantrine were detected in all hair samples, with quantifiable levels in five cases. Artemether and its active metabolite dihydroartemisinin were undetectable. No study participants developed recrudescence.

**Conclusions:**

This study demonstrates that lumefantrine and its metabolite can be detected in hair weeks after treatment, suggesting hair analysis may serve as a retrospective tool to assess drug exposure in recrudescent malaria cases. The absence of artemether and dihydroartemisinin was likely due to their short half-lives, preventing incorporation into hair. A larger study is warranted to evaluate correlations between lumefantrine hair and plasma concentrations. If validated, this approach could aid in distinguishing inadequate drug exposure from reduced parasite susceptibility to AL, optimizing malaria treatment strategies.

## Highlights

• (Desbutyl-)lumefantrine is detectable in hair after artemether-lumefantrine treatment.
• Artemether and its metabolite were undetectable, likely due to their short half-lives.
• Hair analysis may serve as novel tool to retrospectively assess drug exposure.

## 1 Introduction

According to international guidelines, the first-line treatment for acute uncomplicated *Plasmodium (P.) falciparum* malaria is artemether-lumefantrine (AL; brand names Riamet®, Co-artem®), achieving parasite clearance in >95 % of patients [1]. However, 2–14 % of cases develop recrudescence of falciparum malaria (clinical recurrence due to treatment failure) in the weeks after treatment [[2], [3], [4], [5], [6], [7], [8], [9], [10], [11]]. A large pooled analysis of pharmacokinetic-pharmacodynamic data from AL-treated patients, showed that a lumefantrine plasma concentration threshold of ≥200 ng/ml at Day 7 of AL initiation corresponds to a >98 % cure rate [12]. In malaria patients in endemic settings, recrudescence has been associated with high parasitaemia and inadequate Day 7 lumefantrine levels (<200 ng/mL), which may be caused by poor adherence or malabsorption, as it may occur when AL is not taken with a fatty meal [12]. Other proposed factors that may account for recrudescence include a lack of (partial) immunity and decreased susceptibility of *P. falciparum* to AL [4,10,13,14], in particular to the long-acting partner drug of the artemisinin compound, the 4-aminoquinoline lumefantrine (the same phenomena are observed with other artemisinin-based combination therapies [ACT]; we focus here on AL which is the most widely-used ACT for imported malaria). Pinpointing the cause of recrudescence retrospectively is challenging, as blood sampling is currently advised at two and four weeks after AL initiation, when lumefantrine levels may be undetectable, even if they were therapeutic during the first days to week(s). Routine Day 7 blood sampling for all falciparum malaria cases seems impractical, given that recrudescence occurs in only a minority of patients. An alternative approach could be to measure drug levels by hair analysis in patients who experience recrudescence, as hair sampling offers a non-invasive, retrospective measure of drug exposure over weeks to months, allowing clinicians to better differentiate between treatment failure due to inadequate drug exposure versus decreased parasite susceptibility. Hair analysis is already being applied to retrospectively monitor therapeutic levels of HIV and tuberculosis drugs [[15], [16], [17]], and was also recently used to assess adherence to atovaquone/proguanil (AP) and mefloquine malaria chemoprophylaxis in travellers [18]. To our knowledge, no studies have been conducted that attempt to measure artemether and/or lumefantrine concentrations in hair. We hypothesise that AL, and in particular lumefantrine with its elimination half-life of 4–6 days (as compared to the short half-lives of artemisinin and its active metabolite of 2 h), penetrate the hair matrix [19]. If that was the case, and if hair concentrations correlated with plasma concentrations, hair analysis could serve as a novel method to retrospectively assess the adequacy of drug exposure during the AL-course and discriminate between resistance and malabsorption when recrudescence occurs. The objective of this proof-of-concept was to assess whether artemether and lumefantrine, and their respective metabolites dihydroartemisinin and desbutyl-lumefantrine, could be detected and quantified in hair of falciparum malaria patients who completed an AL treatment course.

## 2 Methods

### 2.1 Study population

Adult Amsterdam UMC patients, who were diagnosed with falciparum malaria and completed an AL treatment course between January 1 and October 1, 2024, were asked to participate. Treatment consisted of six doses of 80 mg artemether and 480 mg lumefantrine (four tablets, each containing 20 mg artemether and 120 mg lumefantrine) administered on diagnosis and at 8, 24, 36, 48, and 60 h [1,19]. Patients were instructed to take all tablets with a fatty meal, and the first dose was observed. Only patients with brown and black hair were eligible for this study, to increase the potential to detect drug levels, as pigmented hair has been shown to enhance drug incorporation and binding [20]. Patients using permanent cosmetic hair treatments, such as bleaching and dyeing, were excluded as they may reduce drug content in hair [21]. Additionally, participants were asked to refrain from haircuts during the study period.

### 2.2 Study procedures

A lock of hair, approximately pencil-thick, was cut from the scalp at the posterior vertex region four weeks after AL initiation (Day 28), when falciparum malaria patients visited the Amsterdam UMC as part of standard care, to measure parasitaemia to assess recrudescence. Hair for drug testing is typically taken from the posterior vertex region, as it has the largest percentage of follicles in growth phase, and exhibits the most uniform and consistent growth pattern [22]. Hair samples were secured in foil, with the root end marked with an elastic band, and stored in an envelope in a dry, dark environment at room temperature. Hair samples were analysed by liquid chromatography with tandem mass spectrometry (LC-MS/MS) to detect artemether and lumefantrine, and their respective metabolites dihydroartemisinin and desbutyl-lumefantrine. Furthermore, attempts were made to quantify drug concentrations. A detailed description of the methodology of the analysis will be submitted elsewhere (manuscript in preparation). The following data from the electronic patient file (EPD; EPIC) was collected in an online case report form (Castor EDC: https://www.castoredc.com/): age, sex, total bodyweight, height, country visited, malaria immune status, malaria treatment, date of first and last AL tablet, missed AL tablets, date of hair sample collection, and occurrence of recrudescence. Data were presented in counts and percentages and median and interquartile ranges (IQR).

### 2.3 Ethical considerations

The medical ethical committee of the Amsterdam UMC approved the study and declared that the study does not fall within the scope of the Dutch Medical Research Involving Human Subject Act (reference number 2023.0815). Informed consent was obtained from all participants.

## 3 Results

Hair samples were collected from seven patients who were treated for falciparum malaria. Inadvertently, one patient was included who was treated with AP instead of AL. All other patients had been treated with a full course of AL and reported full compliance. The median age of participants was 50 years (IQR: 36–56). Five of six cases were male (83 %), and all were black with Afro-textured hair. The median time between start of AL treatment and hair sampling was 28 days (IQR: 28–36). No participants had developed *P. falciparum* recrudescence. Demographics of the study participants and the study results are provided in Table 1.

**Table 1:** Demographic characteristics of study participants and study results.

Case	Age	Sex	TBW	BMI	Country visited	Malaria immunity	Treated with iv artesunate	Start AL to hair sample	Artemether	DHA	Lumefantrine level range	DBL
1	45	Male	87	29.41	Ghana	Non-immune	No	28 days	Not detectable	Not detectable	52–206 ng/g	Detectable
2	36	Male	105	30.35	Kenya	Non-immune	Yes, 2 doses	36 days	Not detectable	Not detectable	16–63 ng/g	Detectable
3	29	Female	66	20.83	Guinea + Sierra Leone	Non-immune	No	28 days	Not detectable	Not detectable	53–447 ng/g	Detectable
4	68	Male	105	NR	Sierra Leone	Non-immune	No	28 days	Not detectable	Not detectable	Detectable	Detectable
5	56	Male	104	NR	Ghana	Non-immune	Yes, 2 doses	27 days	Not detectable	Not detectable	11–50 ng/g	Detectable
6	55	Male	88	NR	Ghana	Non-immune	No	36 days	Not detectable	Not detectable	23–78 ng/g	Detectable

None of the participants missed any tablets. All patients resided in the Netherlands and travelled to Africa to visit family. Definition non-immune = residence in a non-endemic area for three years or longer, and without a history of malaria in the past three years. NR = not reported; NA = not applicable; TBW = total body weight (in kilograms); BMI = body mass index (in kilograms per m^2^); iv = intravenous; AL = artemether/lumefantrine; DHA = dihydroartemisinin; DBL = desbutyl-lumefantrine.

Lumefantrine and desbutyl-lumefantrine concentrations were both detected in the hair samples of all six participants taking AL. As this was a first explorative analysis, not designed for quantification of drug levels, no exact concentrations could be determined. However, ranges of lumefantrine concentration could be determined in five of six cases (83 %); and ranged between 11 and 447 ng per gram of hair. No estimates could be determined for desbutyl-lumefantrine as hair concentrations were below the lowest calibration standard (that equals to 1 ng per millilitre plasma). Artemether and dihydroartemisinin were both not detected in any of the hair samples. The hair sample of the patient who took AP was already analysed when we discovered he was inadvertently included. As expected, artemether dihydroartemisinin, lumefantrine, and desbutyl-lumefantrine were undetectable in this patient.

## 4 Discussion

The aim of this proof-of-concept study was to assess if artemether and/or lumefantrine, and their respective metabolites dihydroartemisinin and desbutyl-lumefantrine, could be detected and quantified in hair in falciparum malaria patients who completed an AL treatment course.

Both lumefantrine and desbutyl-lumefantrine penetrated the hair matrix and could be detected one month after AL initiation. However, artemether and its active metabolite dihydroartemisinin could not be detected in hair. One explanation for this may be that artemether and dihydroartemisinin are so rapidly cleared from the plasma (terminal half-life of around 2 h) that the capillaries of hair follicles were not exposed long enough for these drug concentrations to penetrate the hair matrix [19]. Samples were handled in accordance with recommendations from the Society of Hair Testing [22], and the explorative analysis was validated in a limited manner in accordance with the ICH guideline M10 on bioanalytical method validation [23]. Therefore, it is unlikely that any flaws in sample handling or analysis account for the inability to detect artemether and dihydroartemisinin.

Nevertheless, our aim was to assess if hair analysis could be applied as a novel method to retrospectively assess the adequacy of drug exposure when recrudescence occurs. Inadequate lumefantrine and desbutyl-lumefantrine concentrations will result in recrudescence, whilst inadequate artemether and dihydroartemisinin concentrations cause delayed parasite clearance [24]. Thus, for this purpose, it was crucial to detect and quantify lumefantrine and desbutyl-lumefantrine concentrations, and less relevant to detect artemether and dihydroartemisinin in hair. Therefore, the successful detection and quantification of lumefantrine and its metabolite in hair are clinically meaningful for this purpose, whereas the lack of artemether and dihydroartemisinin detection is less critical.

A notable advantage of using hair for monitoring drug levels, instead of blood or urine, is that drug exposure can be quantified over a longer period (weeks to months) after drug intake, enabling sampling when the need arises (on recrudescence, i.e. weeks after treatment), rather than through proactive blood draws on treatment initiation. Additionally, the sampling of hair is a less invasive procedure than venepunctures.

However, for hair concentrations to be of clinical value, a correlation between hair and plasma concentration is required, as plasma concentrations correspond to the risk of recrudescence [12]. Correlations have been established for various drugs, such as carbamazepine, phenobarbital and amitriptyline [25]. The lumefantrine plasma concentration on Day 7 has been shown to correlate with the area under the concentration time curve and the treatment response [12]. A larger follow-up study will be performed to assess if lumefantrine and desbutyl-lumefantrine hair concentrations are correlated with plasma concentrations. If so, hair analysis may be applied as a novel methodology to retrospectively assess the adequacy of drug exposure when recrudescence occurs, and to discriminate from reduced susceptibility of the *P. falciparum* to AL.

## 5 Conclusion

This study demonstrated that lumefantrine and its metabolite are detectable in hair one month after treatment, supporting the feasibility of hair analysis as a retrospective measure of drug exposure in falciparum malaria patients. The absence of artemether and dihydroartemisinin was likely due to their short half-lives, preventing incorporation into hair. Future research should focus on establishing the relationship between (desbutyl-)lumefantrine concentrations in hair and plasma, which is essential for clinical application. If validated, hair analysis could become a valuable non-invasive tool to differentiate between insufficient drug exposure and reduced parasite susceptibility, ultimately informing treatment decisions and improving malaria control.

## CRediT authorship contribution statement

**Jenny L. Schnyder:** Writing – original draft, Project administration, Methodology, Investigation, Formal analysis, Conceptualization. **Marloes Vos-van der Meer:** Writing – review & editing, Project administration, Methodology, Investigation, Formal analysis, Conceptualization. **Reinier M. van Hest:** Writing – review & editing, Supervision, Methodology, Investigation, Conceptualization. **Ron Mathot:** Writing – review & editing, Supervision, Methodology, Investigation, Conceptualization. **Patricia Schlagenhauf:** Writing – review & editing, Conceptualization. **Hanna K. de Jong:** Writing – review & editing, Supervision, Methodology, Investigation, Conceptualization. **Martin P. Grobusch:** Writing – review & editing, Supervision, Methodology, Investigation, Conceptualization.

## Funding

No funding was received for this study.

## Declaration of competing interest

The authors declare that they have no known competing financial interests or personal relationships that could have appeared to influence the work reported in this paper.
